# Multidomain cognitive impairment in non-hospitalized patients with the post-COVID-19 syndrome: results from a prospective monocentric cohort

**DOI:** 10.1007/s00415-022-11444-w

**Published:** 2022-11-23

**Authors:** Ann-Katrin Schild, Yasemin Goereci, Daniel Scharfenberg, Kim Klein, Joachim Lülling, Dix Meiberth, Finja Schweitzer, Sophie Stürmer, Philip Zeyen, Derya Sahin, Gereon R. Fink, Frank Jessen, Christiana Franke, Oezguer A. Onur, Josef Kessler, Clemens Warnke, Franziska Maier

**Affiliations:** 1 Department of Psychiatry, Faculty of Medicine, University Hospital Cologne, University of Cologne, Cologne, Germany; 2 Department of Neurology, Faculty of Medicine, University Hospital Cologne, University of Cologne, Cologne, Germany; 3 German Center for Neurodegenerative Diseases (DZNE), Bonn, Germany; 4 Institute of Neuroscience and Medicine (INM-3), Cognitive Neuroscience, Research Centre Jülich, Jülich, Germany; 5 Excellence Cluster on Cellular Stress Responses in Aging-Associated Diseases (CECAD), University of Cologne, Cologne, Germany; 6 Department of Neurology with Experimental Neurology, Charité-Universitätsmedizin Berlin, Corporate Member of Freie Universität Berlin, Humboldt-Universität zu Berlin, and Berlin Institute of Health, Berlin, Germany

**Keywords:** Cognitive deficits, Neurocognitive disorder, Subjective deficits, Neuropsychology, SARS-CoV-2, Post-COVID-19 syndrome

## Abstract

**Background:**

A fraction of patients with asymptomatic to mild/moderate acute COVID-19 disease report cognitive deficits as part of the post-COVID-19 syndrome. This study aimed to assess the neuropsychological profile of these patients.

**Methods:**

Assessment at baseline (three months or more following acute COVID-19) of a monocentric prospective cohort of patients with post-COVID-19 syndrome. Multidomain neuropsychological tests were performed, and questionnaires on depression, anxiety, fatigue, sleep, and general health status were administered.

**Results:**

Of the 58 patients screened, six were excluded due to possible alternative causes of cognitive impairment (major depression, neurodegenerative disease). Of the remaining 52 individuals, only one had a below-threshold screening result on Mini-Mental State Examination, and 13 scored below the cut-off on Montreal Cognitive Assessment. Extended neuropsychological testing revealed a neurocognitive disorder (NCD) in 31 (59.6%) participants with minor NCD in the majority of cases (*n* = 26). In patients with NCD, the cognitive domains learning/memory and executive functions were impaired in 60.7%, complex attention in 51.6%, language in 35.5%, and perceptual-motor function in 29.0%. Cognitive profiles were associated with daytime sleepiness but not with depression, anxiety, sleep quality, total general health status, or fatigue.

**Conclusion:**

Neurocognitive impairment can be confirmed in around 60% of individuals with self-reported deficits as part of post-COVID-19 syndrome following a mild acute COVID-19 disease course. Notably, screening tests cannot reliably detect this dysfunction. Standard psychiatric assessments showed no association with cognitive profiles. Longitudinal studies are needed to further evaluate the course of neurocognitive deficits and clarify pathophysiology.

**Supplementary Information:**

The online version contains supplementary material available at 10.1007/s00415-022-11444-w.

## Introduction

After the acute phase of infection with severe acute respiratory syndrome coronavirus 2 (SARS-CoV-2), many patients continue to report a broad variety of symptoms comprising pulmonary, cardiovascular, or gastrointestinal symptoms, often worsening after physical activity [1–3]. Many individuals also complain about neuropsychiatric symptoms, particularly cognitive impairment [4, 5]. The mechanism of how coronavirus disease 2019 (COVID-19) leads to cognitive impairment is still largely elusive, but it is most likely multifactorial [6, 7]. One known factor is mechanical ventilation during severe COVID-19 [8]. However, cognitive impairment after COVID-19 is not restricted to hospitalized patients. In a large population-based study, individuals with preceding self-reported COVID-19 performed significantly worse in online cognitive testing [9]. Smaller studies also revealed deficits after mild COVID-19 using remote or in-person cognitive testing [10–14]. Importantly, cognitive dysfunction after asymptomatic to moderate COVID-19 was detected in individuals with self-reported deficits and those with no subjective symptoms [15]. In a review article including 12 studies collecting test data on 1000 patients, the proportion of individuals who recovered from COVID-19 with cognitive impairment ranged from 15 to 80% [16]. This wide range can be explained by differences in sample characteristics (study size; patient selection; disease severity; time from acute COVID-19 to cognitive testing; remote or in-person testing; and test batteries), impeding the comparison of the findings. Most studies are limited by small sample sizes [11, 14], the application of cognitive screening tests instead of comprehensive tests [10, 13, 14] and heterogeneous samples, neglecting disease severity [11–13], reports of subjective cognitive impairments [9–15] or the time between acute COVID-10 infection to neuropsychological assessment [9, 11, 14). In addition, it is uncertain at present, which cognitive domains are preferentially affected in the cognitive post-COVID-19 syndrome.

Here, we performed a comprehensive in-person standardized assessment of the different cognitive domains of learning and memory, language, executive functions, complex attention, and perceptual-motor function in a well-defined sample of individuals after asymptomatic or mild/moderate acute COVID-19 syndrome who reported a subjective cognitive decline. We restricted our inclusion criteria to those fulfilling the definition of the National Institute for Health and Care Excellence (NICE) for the post-COVID-19 syndrome [17]. Retrospectively, all participants also fulfilled the novel definition of the World Health Organization (WHO) for the post-COVID-19 condition [18]. Additionally, questionnaires and scales regarding depression, anxiety, sleep quality, daytime sleepiness, fatigue, and general health status were administered [19, 20].

## Patients and methods

Patients were recruited from specialized neurological or psychiatric post-COVID-19 outpatient clinics between 03/2021 and 09/2021. The Institutional Review Board of the University of Cologne granted ethical approval (20–1501). The study was registered in the German Clinical Trials Register (DRKS00024434).

Patients learned about the post-COVID-19 program from our institutional website, were referred by the department of infectious diseases, or contacted us directly with various symptoms of post-COVID-19. Eligible for our study were patients older than 18 years after an asymptomatic, mild or moderate confirmed SARS-CoV-2 disease course, who reported cognitive deficits persisting at least three months. Known pre-morbid mild cognitive impairment, dementia, or a history of severe psychiatric or neurological condition within the last two years were exclusion criteria. Written informed consent was obtained from all patients before enrollment.

Demographic information and medical history were obtained using questionnaires or a semi-structured interview. Self-reported symptoms during the acute phase of COVID-19 infection (Supplement Table 1) as well as self-reported post-COVID-19 symptoms that persisted for more than three months before the date of neuropsychological assessment (Supplement Table 2) were recorded retrospectively.

Cognitive screening tests were the Mini-Mental State Examination (MMSE) at the beginning of the neuropsychological assessment and the Montreal Cognitive Assessment (MoCA) at the end [21, 22].

All participants underwent cognitive testing with the full neuropsychological test battery, which was administered by two trained neuropsychologists and extended over 120 min.

The test selection was guided by the cognitive domains defined in DSM-5, covering learning and memory, complex attention, executive functions, language, and perceptual-motor function [23]. The domain of social cognition was not covered to limit test duration. Based on available normative data corrected for age, gender, and level of education, we created two different test sets: one for patients younger than 50 and one for patients at or above the age of 50 years. We ensured that tests measuring the same cognitive function (e.g., VLMT and CERAD word list for testing verbal memory) had the same test sequence making both test sets comparable. All information on neuropsychological tests is listed in Supplement Table 3.

To assess symptoms of depression and anxiety, we used the Hospital Anxiety and Depression Scale [24]. To assess fatigue, we applied the Fatigue Severity Scale [25]. Sleep quality was measured with the Pittsburgh Sleep Quality Index [26], and daytime sleepiness was assessed with the Epworth Sleepiness Scale [27]. We measured general health status with the Short-Form-36 Health Survey [28]. We created a total general health score by computing the unweighted mean of the domain-specific values of the SF-36 (range 0–100, with lower values indicating worse health ratings).

The following DSM-5 manual guided classification with regard to cognitive performance was applied:

1. No NCD (NoNCD): none or one test score indicates a cognitive deficit defined by a performance of at least one standard deviation (SD) below the mean of the norm.
2. Minor NCD (MinNCD): at least two test scores indicate a cognitive deficit (between one and two SD below the mean of the norm).
3. Major NCD (MajNCD): at least two test scores indicate a severe cognitive deficit (at least two SD below the mean of the norm).

The cognitive domains of learning and memory, complex attention, and executive functions each contained five sub-scores, the domain of language contained two, and the domain of perceptual-motor function one sub-score.

Five domain composite scores (DCS) were established (DCS learning and memory, DCS complex attention, DCS executive functions, DCS perceptual-motor function, and DCS language). For this purpose, we transformed all cognitive test scores (T-values, percentiles, etc.) into z-values. After the transformation, we created the domain composite scores (DCS) for each participant with R package multicon [29], which computes the unweighted mean of all z-values for the respective cognitive domain. The DCS were not computed if more than 20% of the data were missing. While this was the case in two patients in the DCS learning and memory, we were able to compute all other DCS for every patient. Supplement Table 3 gives an overview of the different cognitive domains and the tasks of the two different test sets.

Moreover, we created a global cognitive composite score (GCCS) for each patient by computing the unweighted mean of the five DCS.

We conducted statistical analyses with R [30] (Version 4.0.5). All statistical tests were performed at an alpha level of 0.05. We performed one-way ANOVAs comparing demographic variables (age, years of education, time between infection and neuropsychological assessment), psychiatric and general health scores (raw scores of HADS, FSS, PSQI, ESS, and SF-36; Table 1), cognitive screening tests (MMSE and MoCA; Table 1), DCSs, and the GCCS between the different groups of cognitive impairment (No NCD, Minor NCD, Major NCD) without adjusting for multiple comparisons due to the exploratory nature of the study. If Levene’s tests indicated heterogeneity of variances, we used Welch’s ANOVA. If the ANOVA yielded significant results, we calculated Tukey’s HSD for variables with homogeneity of variances and Games–Howell tests for variables with heterogeneity of variances for post hoc comparisons. To determine if there was an association between gender as well as the two different test sets (depending on age) and the three groups of cognitive impairment, we performed Fisher’s exact test.

**Table 1:** Demographics, cognitive screening tests, different DCS, GCCS, and additional assessments by level of cognitive impairment

	No NCD *N* = 21 (40.38%)	Minor NCD *N* = 26 (50%)	Major NCD *N* = 5 (9.62%)		*P* value	
	*N* (%)	*N* (%)	*N* (%)			
*Demographics*						
Male	8 (38.10)	13 (50.00)	0 (0.00)		0.125^a^	
	**Mean (SD)**	**Mean (SD)**	**Mean (SD)**	***F***	***P* value**	**Direction**
Age, *years*	46.71 (7.9)	46.23 (11.9)	46.6 (12.4)	0.01	0.987	
Education, *years*	15.86 (2.1)	15.58 (2.8)	13.8 (1.8)	1.45	0.244	
Premorbid IQ	109.20^b^ (9.9)	107.77 (9.0)	102.40 (17.3)	1.40	0.242	
Days after infection—NPA	238.95 (129.3)	237.8 (101.2)	291.2 (177.6)	0.43	0.651	
*Cognitive screenings*						
MMSE	29.67 (0.6)	29.35 (0.8)	28.40 (1.7)	2.27 ^c^	0.154	
MoCA	27.3^b^ (2.9)	26.88 (2.2)	23.20 (4.8)	5.30	0.026	NoNCD = MinNCD > MajNCD^d^
*Composite scores*						
GCCS	0.38 (0.3)	– 0.11 (0.4)	– 0.93 (0.5)	28.2	< 0.001	NoNCD > MinNCD > MajNCD^d^
DCS Learning and memory	0.35 (0.5)	– 0.14 (0.6)	– 1.30 (0.6)	15.69	< 0.001	NoNCD > MinNCD > MajNCD^d^
DCS Complex attention	0.55 (0.3)	0.00 (0.7)	– 1.00 (0.5)	26.21^c^	< 0.001	NoNCD > MinNCD > MajNCD^e^
DCS Executive functions	0.37 (0.5)	0.01 (0.6)	– 0.82 (0.8)	10.06	< 0.001	NoNCD = MinNCD > MajNCD^d^
DCS Language	0.35 (0.7)	– 0.21 (0.6)	– 0.61 (1.2)	5.72	0.006	NoNCD > MinNCD = MajNCD^d^
DCS Perceptual-motor function	0.29 (0.6)	– 0.20 (0.8)	– 0.95 (1.1)	9.01	0.005	NoNCD = MinNCD; MinNCD = MajNCD; NoNCD > MajNCD^d^
*Psychiatric scales*						
HADS Depression	6.33 (4.5)	6.36^f^ (3.8)	6.60 (3.3)	0.00	0.991	
HADS Anxiety	6.33 (3.0)	7.12^f^ (4.0)	7.00 (3.2)	0.29	0.749	
FSS	42.76 (14.3)	42.27 (11.3)	45.40 (18.9)	0.12	0.891	
PSQI	8.00 (4.2)	8.76^f^ (3.9)	9.00 (4.5)	0.24	0.784	
ESS	9.95 (5.3)	8.12 (5.8)	15.20 (5.8)	3.48	0.039	NoNCD = MinNCD; NoNCD = MajNCD; MajNCD > MinNCD^d^
SF-36 Total	45.92^b^ (17.0)	43.85 (13.3)	39.90^ g^ (8.1)	0.31	0.733	

*NCD* Neurocognitive disorder, *Premorbid IQ* Verbal intelligence to estimate premorbid IQ was assessed with the Mehrfachwahl-Wortschatztest Version B (MWT-B) [31] or Wortchatztest (WST) [32]; *days infection – NPA* days between infection and neuropsychological assessment, *MMSE* Mini-Mental State Examination (cut-off < 27 impaired cognition [22, 33]); *MoCA* Montreal Cognitive Assessment (cut-off ≤ 25 impaired cognition [21]); *GCCS* Global cognitive composite score; *DCS* Domain composite score; Hospital Anxiety and Depression Scale (max. score: 21, cut-off score > 10) [24]; Fatigue Severity Scale was applied (max. score 63, score > 36 indicative for fatigue syndrome [25]; Pittsburgh Sleep Quality Index (max. score 21, score 6–10 = poor sleep quality, > 10 chronic sleep disturbance [26]; Epworth Sleepiness Scale (max. score 24, 8–10 indicative for elevated day time sleepiness, > 10 strongly elevated day time sleepiness [27]); Short-Form-36 Health Survey (range 0–100, higher value reflects better health status [28])

^a^Fisher’s exact test, ^b^*N* = 20, ^c^Welch F-Test for unequal variances, ^d^Tukey’s HSD, ^e^Games–Howell Test, ^f^*N* = 25 g N = 4

## Results

### Demographics

58 patients were included in the study with a mean of 243 days following COVID-19 onset (range 92–554 days). Six patients were excluded (four due to a major depressive episode and two due to a neurodegenerative disease), resulting in 52 patients (21 male) with a mean age of 46.5 years (Table 1). The level of education was high with a mean of 15.5 years (± 2.5). No patient had a lexical IQ score below the normative range. All patients included in this study had COVID-19 before vaccination was available in Germany.

At study inclusion, patients reported memory impairment and concentration deficit as well as fatigue as main post-COVID-19 symptoms (94.2%; 76.9%), followed by headache (38.5%), sleep disorder, and limb pain/myalgia/ arthralgia (both 21.2%) (Supplement Table 2).

### Assessment

The mean score of the MMSE was 29.7 not indicating cognitive impairment. Only one patient had an MMSE score of 26, which provides evidence for mild cognitive impairment (cut-off < 27 impaired cognition) [33].

The MoCA did not show evidence for cognitive impairment at the group level with a mean of 27.3 points. At the individual level, 13 patients scored in the range, which indicates cognitive impairment (cut-off ≤ 25 impaired cognition) [21].

The neuropsychological tests revealed that 31 (59.6%) patients fulfilled criteria of NCD, while 21 (40.4%) showed no measurable cognitive impairment. Of all patients with NCD, 25 (83.9%) were classified with minor NCD, and 5 (16.1%) with major NCD (Table 1). Most NCD patients (*n* = 27; 87.1%) had multidomain cognitive impairment.

The neuropsychological profiles did not differ between patients tested with the test battery for younger (*n* = 35) and older participants (*n* = 17) (*p* = 0.56).

ANOVA revealed no significant differences in age, years of education, days between infection and neuropsychological assessment, and premorbid IQ between groups of participants qualifying for minor or major NCD or without objective impairment (all *p* > 0.242). There was also no difference in gender distribution between the three groups of different cognitive impairments (*p* = 0.125).

There were no group differences in the MMSE scores between the three groups. With regard to the MoCA, there was a significant group effect (*p* = 0.026). Post hoc tests showed that patients with major NCD scored lower than patients without NCD or with minor NCD, while there was no significant difference between the latter two groups.

**Fig. 1:**
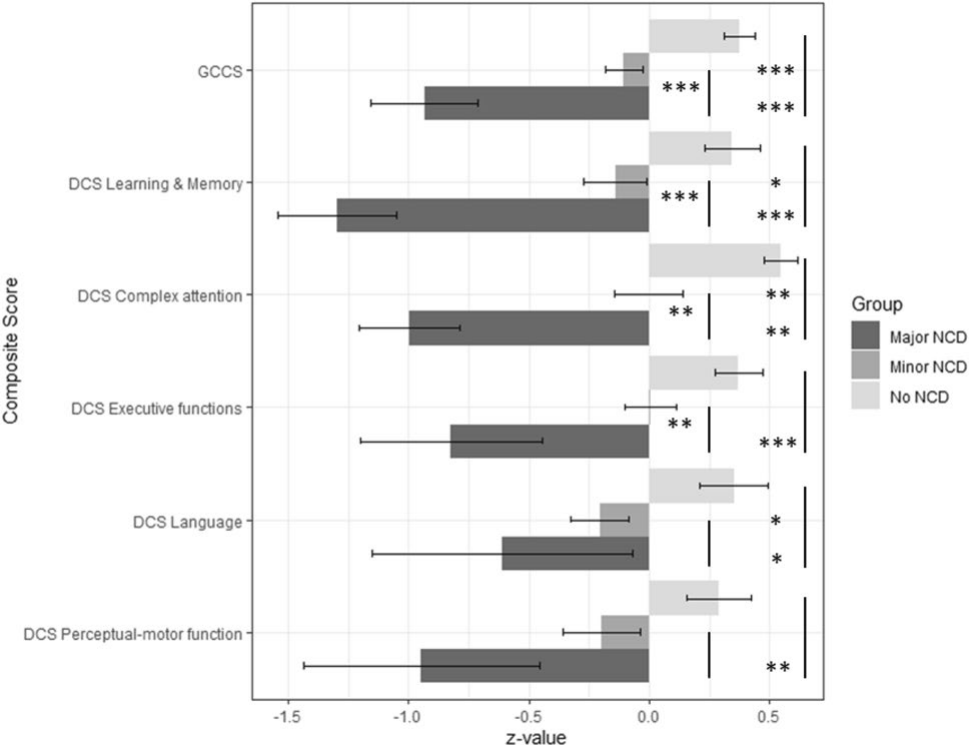
Results of cognitive performance in different domains (DCS) and global cognition (GCCS), depending on the level of cognitive impairment (no NCD, minor NCD, major NCD). *GCSS* global composite score, *DCS* domain composite score, *NCD* neurocognitive disorder; error bars depict standard error of mean *z*-value. *p < 0.05. **p < 0.01. ***p < 0.001

GCCS showed significant differences between the three groups (*p* < 0.001). Patients without NCD performed better than patients with minor NCD. Both groups (no NCD and minor NCD) showed better performance than patients with major NCD (Fig. 1).

Moreover, for each DCS, a significant group effect was found. Post hoc tests revealed for DCS learning and memory (*p* < 0.001) and DCS complex attention (*p* < 0.001) the same group differences as for the GCCS. For DCS executive functions, performance between groups of no and minor NCD did not differ, but was better than in patients with major NCD (*p* < 0.001). For DCS language, post hoc tests revealed a better performance in patients without NCD than in both NCD groups (*p* = 0.006). Finally, for DCS perceptual-motor function patients without NCD were significantly better than patients with major NCD (*p* = 0.005) (Fig. 1).

The additional assessments (Table 1) revealed the mean values for depression and anxiety at the borderline to clinically relevant severity of symptoms [34] and also revealed no group differences. The mean fatigue score was 41.8 and above the proposed threshold of clinical relevance (36), and PSQI and ESS showed poor sleep quality and elevated daytime sleepiness. The mean total general health status was 45.9, indicating an average status (mean 50 ± 10). Of all assessments, only daytime sleepiness showed a significant effect between groups with higher scores in major compared to minor NCD (*p* = 0.039).

## Discussion

The post-COVID-19 syndrome is a multifaceted condition, which may affect cognition [4, 19]. We focused on patients reporting cognitive impairment among other symptoms at least three months after an asymptomatic to mild/moderate acute COVID-19 disease course. We conducted an extensive neuropsychological assessment, including cognitive screening tests and psychiatric assessments in 52 patients. In 60% of the patients, subjective cognitive impairment was objectified, of which the majority (around 87%) displayed an impairment in more than one domain. Significant group differences between patients with no, minor, or major NCD were found in all cognitive domains and global cognitive performance as expressed by the GCCS. Neither depression, anxiety, fatigue, sleep quality, nor total general health status differed significantlybetween the groups. Only daytime sleepiness was reported significantly less in patients with minor NCD compared with patients with major NCD.

The patient demographics of our study cohort were similar to other studies on the post-COVID-19 syndrome, with an average age of 46.5 years and a high educational level (15.9 years [9, 12, 19, 35, 36]).

The MMSE that is usually administered for the detection of dementia was not sufficiently sensitive to detect cognitive impairment in our patients, confirming findings by Mattioli et al. [37]. It showed a ceiling effect with small standard deviation and range. The target population of such screening tests usually is older than the patients included in this study which might serve as explanation [38]. The MoCA is more sensitive for the detection of mild cognitive impairment [39]. It was frequently administered in post-COVID-19 patients, where it was able to detect impairment [40, 41]. The MoCA scores in our study indicated impairment in 13 patients. However, the comprehensive neuropsychological assessments revealed impairment in 31 patients indicating still limited sensitivity of the MoCA for post-COVID cognitive dysfunction.

Our findings show cognitive impairment across all cognitive domains in line with other studies reporting deficits in learning and memory, language, or executive functions [11, 12, 14, 16, 42, 43]. However, to our knowledge, only one study has assessed all of the five domains within the same sample and collected additional information on psychiatric variables [44]. In that study, neuropsychological deficits were detected in all domains of cognition (except ideomotor praxis) in patients six to nine months after severe, moderate or mild SARS-CoV-2 infection (15 patients per group). It is critical to point out that we required self-reported cognitive impairment as the inclusion criterion in our study. Therefore, a direct comparison between both studies is limited.

A recent systematic review and meta-analysis of 43 studies on cognition in post-COVID confirmed the high prevalence of cognitive symptoms in the post-COVID-19 syndrome with over one-fifth of subjects showing impairment twelve or more weeks after disease onset [35]. Whether vaccination influences the risk and severity of cognitive post-COVID-19 syndrome needs to be investigated in future studies.

The reasons for cognitive impairment in patients after COVID-19 disease still remain largely elusive. In a previous CSF study of patients with post-COVID-19, we did neither detect SARS-CoV2-RNA nor antibodies suggestive of viral persistence or direct infection of the CNS as an explanation for post-COVID-19 [45]. However, evidence of disturbed endothelial function and blood–brain barrier leakage has been observed in CSF studies during acute COVID-19 by others [46]. This may be associated with an increase in pro-inflammatory cytokines in CSF and blood and could trigger auto-inflammation and autoimmunity as a cause of cognitive dysfunction, noticed in a fraction of individuals during acute COVID-19. Furthermore, a role for anti-idiotype antibodies following infection or vaccination has been proposed recently [47, 48], but experimental evidence is still lacking. Since no specific target of autoimmunity has yet been defined and studies confirming efficacy of immune therapy for post-COVID-19 have not been reported, immune-mediated pathogenesis still remains hypothetical. Psychological approaches like the recently proposed network perspective on neuropsychiatric and cognitive symptoms of the post-COVID-19 syndrome in combination with neurological mechanisms may contribute to explain the variety and persistence of such symptoms after COVID-19 [49].

The patients in our study showed elevated fatigue, poor sleep quality, and increased daytime sleepiness as well as screening scores for depression and anxiety at the borderline to clinical significance at the group level. However, we did not find an association of these assessments with cognition with the exception of higher daytime sleepiness in those with major NCD. Other studies in post-COVID-19 syndrome after mild COVID-19 infection also failed to show associations between cognition and fatigue or depression [14]. This is in contrast with one other study that reported correlations between global cognitive impairment and anxiety and depression in previously hospitalized patients [50].

Importantly, our study included a highly selected group of participants that all reported subjective cognitive impairment or fatigue following an asymptomatic to mild/moderate acute COVID-19 disease course. In this patient population, we observed objective cognitive impairment only in 60%, while 40% showed normal cognitive performance. Patients without objective cognitive impairment did not show higher scores in psychiatric or other assessment variables, which might serve as an alternative explanation for the subjective cognitive impairment. Patients with only subjective impairment may have higher premorbid capacities, which would require higher thresholds to define impairment at an individual level or which may allow compensation during testing. Also other aspects, like increased self-reflection or specific personality traits in those with subjective impairment only, may contribute to this finding. Data from a large cohort in France further suggest that persistent symptoms may be associated more with the belief in having had a SARS-CoV-2 infection rather than with actually confirmed COVID-19 [51]. In one study, a comparison of cognitive performance between patients with and without subjective cognitive complaints after hospital discharge did not reveal significant performance differences, although anxiety and depression substantially differed between the groups [11]. Lack of awareness of cognitive impairment (i.e., anosognosia) has recently been shown to discriminate between clinical phenotypes of the post-COVID-19 syndrome [44].

The strengths of our study are the rigid inclusion criteria regarding the severity of acute COVID-19 disease, the definition of the post-COVID-19 syndrome, and the homogenous sample that reported cognitive impairment. Moreover, five out of six cognitive domains of the DSM-V were systematically assessed. Psychiatric and other features were additionally collected. A limitation to the study is the lack of a matched control group (persons that never had COVID-19, persons with post-COVID-19 syndrome who do not report cognitive impairment or persons who had a severe or critical acute COVID-19 disease course), and the exploratory cohort of limited size. Indeed, the frequency of around 60% of NCD seen in our cohort using a sensitive testing approach should not be generalized to the overall post-COVID-19 patient population: We studied a subpopulation with self-reported cognitive deficits as inclusion criterion, likely overestimating its frequency in the overall post-COVID-19 patient population. Thus, our study should not be considered a study that assesses prevalence of NCD in post-COVID-19 syndrome in general. Another limiting factor is the single time-point of measurement. A first study assessing two-time points indicated that, in contrast to other symptoms, cognitive impairment might remain—at least in subjective perception—and may even worsen over time in some subjects [52]. In agreement, a recent meta-analysis did not find a decrease in proportion of subjects with cognitive symptoms at less than six compared to more than six months of follow-ups [35]. However, more and well-designed longitudinal studies are required prior to drawing firm conclusions.

## Conclusions

Our study presents neuropsychological profiles of patients with post-COVID-19 syndrome after asymptomatic or mild to moderate infection. Around 60% of patients who initially reported subjective impairment had deficits that were mostly not detectable with a cognitive screening test. All five cognitive domains tested were affected, and the majority of patients had a multidomain cognitive impairment. We did not find differences in demographics or psychiatric or other scores between the groups with and without objective impairment.

Our detailed characterization of cognitive deficits may help to advance the current understanding and definition of the post-COVID-19 syndrome, which may incorporate classifications of minor and major NCD as done here.

Considering the rigid inclusion criteria, our study has limited generalizability to the entirety of post-COVID-19 patients: Likely, the frequency of confirmed cognitive deficits in post-COVID-19 is far below the 60% reported in our study that included only patients with self-reported cognitive deficits who presented at a specialized post-COVID-19 outpatient clinic. Additionally, overestimation of the frequency of confirmed cognitive deficits in the reported post-COVID-19 subgroup might be endorsed considering the sensitive and extensive testing protocol of the study.

Specific biomarkers to support the definition of the post-COVID-19 syndrome are currently missing. Still, data so far do not suggest that post-COVID-19 is explained by persisting central nervous system infection [45], leaving brain imaging alterations observed largely unexplained [53]. Other possible aspects may include immune-mediated [48, 54], neurodegenerative, hypoxia-related [55, 56] or metabolic changes related [45, 57] causes. Furthermore we should not neglect possible complex multifactorial explanations including psychiatric network perspectives [49].

To address post-COVID-19-associated cognitive deficits over time, large longitudinal studies on the natural course of the subjectively reported and objective neurocognitive deficits with unimpaired patients, who experienced a SARS-CoV-2 infection as a control group are needed. This may help to eventually identify diagnostic and predictive markers for this condition and to understand the underlying pathophysiology.

## Supplementary Information

Below is the link to the electronic supplementary material.

Supplementary file1 (DOCX 56 KB)
