# Supplementary material for: Multidomain cognitive impairment in non-hospitalized patients with the post-COVID-19 syndrome: results from a prospective monocentric cohort
Source: J Neurol. 2022 Nov 23;270(3):1215–23. doi: 10.1007/s00415-022-11444-w (PMC9686246; doi:10.1007/s00415-022-11444-w)
Supplement: Supplementary file 1 — Supplementary file1 (DOCX 56 KB) [file 415_2022_11444_MOESM1_ESM.docx]

**SUPPLEMENT**

**Table S1**

**Self-reported symptoms during the acute phase of COVID-19 infection and frequency of subsequently assessed cognitive classification (N = 51)**

| Symptoms | Reported | Reported in % | noNCD (%) | minNCD (%) | majNCD (%) |
| --- | --- | --- | --- | --- | --- |
| headache | 24 | 46.15 | 9 (37.50) | 15 (62.5) | 0 (0.00) |
| fever | 23 | 44.23 | 7 (30.43) | 13 (56.52) | 3 (13.04) |
| anosmia, ageusia | 23 | 44.23 | 9 (39.13) | 11 (47.83) | 3 (13.04) |
| myalgia, arthralgia | 21 | 40.38 | 9 (42.86) | 10 (47.62) | 2 (9.52) |
| dyspnoea | 14 | 26.92 | 5 (35.71) | 7 (50.00) | 2 (14.29) |
| cough | 13 | 25.00 | 3 (23.10) | 8 (61.54) | 2 (15.38) |
| fatigue | 10 | 19.23 | 5 (50.00) | 2 (20.00) | 3 (30.00) |
| shivering and chills | 9 | 17.31 | 3 (33.33) | 6 (66.67) | 0 (0.00) |
| sore throat | 8 | 15.38 | 1 (12.50) | 6 (75.00) | 1 (12.50) |
| dizziness | 5 | 9.62 | 2 (40.00) | 3 (60.00) | 0 (0.00) |
| diarrhea | 4 | 7.69 | 0 (0.00) | 4 (100.00) | 0 (0.00) |
| subfebrile temperature | 4 | 7.69 | 1 (25.00) | 3 (75.00) | 0 (0.00) |
| nausea | 3 | 5.77 | 1 (33.33) | 1 (33.33) | 1 (33.33) |
| pneumonia | 3 | 5.77 | 1 (33.33) | 1 (33.33) | 1 (33.33) |
| loss of appetite | 2 | 3.85 | 1 (50.00) | 1 (50.00) | 0 (0.00) |
| sinusitis | 2 | 3.85 | 0 (0.00) | 2 (100.00) | 0 (0.00) |
| ear pain | 2 | 3.85 | 1 (50.00) | 1 (50.00) | 0 (0.00) |
| abdominal pain | 2 | 3.85 | 1 (50.00) | 1 (50.00) | 0 (0.00) |
| palpitations | 1 | 1.92 | 1 (100.00) | 0 (0.00) | 0 (0.00) |
| pleuritis | 1 | 1.92 | 0 (0.00) | 0 (0.00) | 1 (100.00) |
| dysesthesia | 1 | 1.92 | 1 (100.00) | 0 (0.00) | 0 (0.00) |

After study inclusion, patients were asked to report retrospectively symptoms during the acute phase of their COVID-19 infection (naming one or more symptom(s) was possible, no structured assessment).

**Table S2**

**Self-reported post-COVID-19 symptoms (N = 52)**

| Symptoms | Reported | Reported in % |
| --- | --- | --- |
| memory impairment/  concentration deficit^a^ | 49 | 94.23 |
| fatigue | 40 | 76.92 |
| headache | 20 | 38.46 |
| sleep disorder | 11 | 21.15 |
| limb pain/ myalgia/ arthralgia | 11 | 21.15 |
| dyspnoea | 8 | 15.38 |
| anosmia/ageusia | 7 | 13.46 |
| visual impairment | 7 | 13.46 |
| dizziness | 6 | 11.54 |
| irritability/ loss of temper | 6 | 11.54 |
| dysesthesia | 6 | 11.54 |
| palpitations | 3 | 5.77 |
| abdominal pain | 2 | 3.85 |
| ear pressure/pain | 2 | 3.85 |
| emotional indifference | 1 | 1.92 |
| hairloss | 1 | 1.92 |
| panic attacks/ anxiety | 1 | 1.92 |
| globus sensation during swallowing | 1 | 1.92 |
| nausea | 1 | 1.92 |
| depression | 1 | 1.92 |

After study inclusion, patients were asked to report post-COVID-19 symptoms that were persistent for more than three months until the day of neuropsychological assessment (naming one or more symptom(s) was possible, no structured assessment).

^a^ Note that the symptom “memory impairment/ concentration deficit” was reported after study inclusion that required post-COVID-19 symptoms of subjective cognitive impairment as an explicit inclusion criterion.

**Table S3**

**Mean z-scores and standard deviations of the different cognitive tasks based on test specific normative data in subjects younger or equal to/older than 50 years of age representing the basis for the aggregated domain composite scores**

| Domain composite score | | | Cognitive task | | Cognitive measure | | | |
| --- | --- | --- | --- | --- | --- | --- | --- | --- |
|  | **Mean (SD)**  **N=52** | |  | **Mean (SD)**  **N=52** | **< 50 years**  **N=35** | **Mean (SD)** | **≥ 50 years**  **N=17** | **Mean (SD)** |
| *Learning & memory* | -0.06 (0.75) | Short term memory | | -0.12 (1.11) | WMS digit span forward^a^ | -0.23 (1.22) | WMS digit span forward^a^ | 0.09 (0.80) |
|  |  | Verbal learning | | 0.01 (1.03) | VLMT Sum rounds 1-5 ^b^ | 0.19 (1.10) | CERAD wordlist sum rounds 1-3^c^ | -0.36 (0.77) |
|  |  | Verbal recall | | 0.00 (0.95) | VLMT round 7 ^b^ | 0.20 (0.90) | CERAD wordlist recall ^c^ | -0.38 (0.97) |
|  |  | Verbal recognition | | 0.09 (1.15) | VLMT recognition ^b^ | 0.28, (1.13) | CERAD recognition ^c^ | -0.26 (1.14) |
|  |  | Visual recall | | -0.33 (1.15) | WMS-IV visual recall II^d^ | 0.02 (1.01) | CERAD visual recall ^c^ | -1.06 (1.12) |
| *Complex attention* | 0.12 (0.72) | Selective attention | | 0.13 (1.15) | TMT-A^e^ | 0.20 (1.04) | TMT-A^e^ | 0.01 (1.36) |
|  |  | Divided attention | | 0.46 (0.99) | TMT-B^e^ | 0.56 (0.87) | TMT-B^e^ | 0.24 (1.20) |
|  |  | Attention performance | | 0.25 (0.97) | FAIR performance score ^f^ | 0.45 (0.96) | FAIR performance score ^f^ | -0.14 (0.90) |
|  |  | Attention quality | | -0.41 (0.77) | FAIR quality score ^f^ | -0.47 (0.76) | FAIR quality score ^f^ | -0.30 (0.82) |
|  |  | Attention continuity | | 0.20 (0.96) | FAIR continuity score ^f^ | 0.36 (0.98) | FAIR continuity score ^f^ | -0.13 (0.86) |
| *Executive functions* | 0.07 (0.64) | Processing speed | | -0.32 (1.09) | LDST ^g^ | -0.30 (1.08) | LDST ^g^ | -0.37 (1.15) |
|  |  | Working memory | | 0.13 (1.22) | WMS span backward^a^ | 0.15 (1.27) | WMS span backward^a^ | 0.10 (1.15) |
|  |  | Stroop read | | -0.13 (0.83) | Stroop read ^h^ | -0.15 (0.93) | Stroop read ^h^ | -0.09 (0.59) |
|  |  | Stroop name | | 0.18 (0.79) | Stroop name ^h^ | 0.17 (0.80) | Stroop name ^h^ | 0.21 (0.81) |
|  |  | Stroop interference | | 0.48 (0.79) | Stroop interference ^h^ | 0.51 (0.78) | Stroop interference ^h^ | 0.42 (0.83) |
| *Perc.-motor function* | -0.07 (0.84) | Visual reproduction | | -0.07 (0.84) | WMS-IV visual recall I^d^ | -0.01 (0.82) | CERAD constructional praxis ^c^ | -0.24 (0.89) |
| *Language* | -0.02 (0.76) | Phonem. word fluency | | -0.13 (-0.99) | RWT s-words ^i^ | -0.26 (0.97) | CERAD s-words ^c^ | 0.15 (1.00) |
|  |  | Semantic word fluency | | 0.09 (1.08) | RWT animals ^i^ | 0.29 (1.12) | CERAD animals ^c^ | -0.32 (0.88) |

^a^ Digit span forward and backward from the Wechsler Memory Scale-Revised (WMS-R) [1]; ^b^ Verbaler Lern- und Merkfähigkeitstest [2]; ^c^ [Consortium to Establish a Registry for Alzheimer's Disease](https://sites.duke.edu/centerforaging/cerad/) (CERAD+) [3,4]; ^d^ Wechsler Memory Scale IV (WMS-IV) visual recall I and II [5]; ^e^ Trail-Making-Test (TMT)-A and –B [6];

^f^ Frankfurter Aufmerksamkeitsinventar (FAIR-2) [7]; ^g^ Letter Digit Substitution Test (LDST) [8]; ^h^ Farbe-Wort-Interferenz-Test (FWIT) [9];^i^ one minute verbal fluency task “s-words” or “animals” [10];^j^ Perc.-motor = Perceptual-motor; Phonem. = Phonematic.

References:

1. Elwood RW (1991) The Wechsler Memory Scale—Revised: psychometric characteristics and clinical application. Neuropsychology review 2:179–201-179–201

2. Lux, S., Helmstaedter, C. & Elger, C. E. (1999) Normierungsstudie zum Verbalen Lern- und Merkfähigkeitstest (VLMT). Diagnostica 45:205–211-205–211

3. Moms JC, Heyman A, Mohs RC et al. (1989) The Consortium to Establish a Registry for Alzheimer's Disease (CERAD). Part I. Clinical and neuropsychological assesment of Alzheimer's disease. Neurology 39:1159. <https://doi.org/10.1212/WNL.39.9.1159>

4. Schmid NS, Ehrensperger MM, Berres M et al. (2014) The Extension of the German CERAD Neuropsychological Assessment Battery with Tests Assessing Subcortical, Executive and Frontal Functions Improves Accuracy in Dementia Diagnosis. Dementia and Geriatric Cognitive Disorders Extra 4:322–334. <https://doi.org/10.1159/000357774>

5. Petermann F, Lepach AC (2012) Wechsler Memory Scale - Fourth Edition, German Edition, 4. Edition. Pearson Assessment, Frankfurt am Main

6. Reitan RM (1958) Validity of the Trail Making Test as an Indicator of Organic Brain Damage. Perceptual and Motor Skills 8:271–276. <https://doi.org/10.2466/pms.1958.8.3.271>

7. Moosbrugger, H. & Oehlschlägel, J. (2011) Frankfurter Aufmerksamkeits-Inventar 2 (FAIR-2). Manual. Hogrefe

8. van der Elst W, van Boxtel MP, van Breukelen GJ et al. (2006) The Letter Digit Substitution Test: Normative Data for 1,858 Healthy Participants Aged 24–81 from the Maastricht Aging Study (MAAS): Influence of Age, Education, and Sex. Journal of Clinical and Experimental Neuropsychology 28:998–1009. <https://doi.org/10.1080/13803390591004428>

9. Bäumler G (1985) Farbe-Wort-Interferenztest (FWIT) nach J.R. Stroop. Hogrefe Verlag für Psychologie

10. Aschenbrenner, S., Tucha, O. & Lange, K. W. (2000) Regensburger Wortflüssigkeits-Test (RWT), 1. Auflage. Hogrefe Verlag für Psychologie
